# The effect of ketogenic diet on behaviors and synaptic functions of naive mice

**DOI:** 10.1002/brb3.1246

**Published:** 2019-03-07

**Authors:** Jie Huang, Yuan‐Quan Li, Cui‐Hong Wu, Yun‐Long Zhang, Shen‐Ting Zhao, Yong‐Jun Chen, Yu‐Hong Deng, Aiguo Xuan, Xiang‐Dong Sun

**Affiliations:** ^1^ Key Laboratory of Neurogenetics and Channelopathies of Guangdong Province and the Ministry of Education of China, School of Basic Medical Sciences Institute of Neuroscience and Department of Neurology of the Second Affiliated Hospital of Guangzhou Medical University Guangzhou China; ^2^ Department of Physiology, School of Basic Medical Sciences Guangzhou Medical University Guangzhou China; ^3^ South China Research Center for Acupuncture and Moxibustion Medical College of Acu‐Moxi and Rehabilitation, Guangzhou University of Chinese Medicine Guangzhou China; ^4^ Department of Clinical Nutrition The Second Affiliated Hospital of Guangzhou Medical University Guangzhou China; ^5^ Guangdong Province Key Laboratory of Psychiatric Disorders Guangzhou China

**Keywords:** ketogenic diet, naive mice, behaviors, synaptic plasticity

## Abstract

**Introduction:**

Beyond its application as an epilepsy therapy, the ketogenic diet (KD) has been considered a potential treatment for a variety of other neurological and metabolic disorders. However, whether KD promotes functional restoration by reducing the pathological processes underlying individual diseases or through some independent mechanisms is not clear.

**Methods:**

In this study, we evaluated the effect of KD on a series of behaviors and synaptic functions of young adult naive mice. Wild‐type C57BL/6J mice at age of 2–3 months were fed with control diet or KD for three months. Body weight and caloric intake were monitored throughout the experiments. We assessed behavioral performance with seizure induction, motor coordination and activity, anxiety level, spatial learning and memory, sociability, and depression. Synaptic transmission and long‐term potentiation were also recorded.

**Results:**

KD‐fed mice performed equivalent to control‐diet‐fed mice in the behavioral tests and electrophysiological assays except exhibiting slower weight gain and increased seizure threshold.

**Conclusions:**

Our results contribute to the better understanding of effects of the KD on physiological behaviors and synaptic functions.

## 1 INTRODUCTION

A ketogenic diet (KD) is diet that is rich in fat, have adequate protein and low carbohydrates content, and known to induce and sustain a ketotic state in the body by producing high levels of ketone bodies (Hallbook, Ji, Maudsley, & Martin, 2012; Boison, 2017). Since the 1920s, the KD has been extensively utilized as a useful strategy to treat refractive epilepsy (Youngson, Morris, & Ballard, 2017). During the last decades, application of KD has also been extended to other neurological or non‐neurological diseases, and demonstrated its beneficial effects (Koppel & Swerdlow, 2018). For example, KD was reported to reduce the anxiety and improve motor behavior in mice with Rett Syndrome (Mantis, Fritz, Marsh, Heinrichs, & Seyfried, 2009); rescue hippocampal memory defects in mice with Kabuki syndrome (Benjamin et al., 2017); exhibit anxiolytic and cognition‐sparing properties or improves motor performance in mouse models of Alzheimer's disease (Brownlow, Benner, D'Agostino, Gordon, & Morgan, 2013; Kashiwaya et al., 2013); improve motor performance in SOD1‐G93A transgenic mice of amyotrophic lateral sclerosis (ALS) (Zhao et al., 2006); delay weight loss in the R6/2 1J mouse model of Huntington's disease (Ruskin et al., 2011); reverse behavioral abnormalities in an acute NMDA receptor hypofunction model of schizophrenia (Kraeuter, Loxton, Lima, Rudd, & Sarnyai, 2015); and reverse diabetic nephropathy, which is a profound diabetic complication (Poplawski et al., 2011).

Despite a variety of beneficial effects of KD on various diseases in animal studies, it is not clear whether KD offers beneficial effects in normal animals. In other words, whether the beneficial effects of KD are restricted to behavioral impairments occurring in diseases or it affects physiological behavioral performance in general is not well understood. Answer to this question is important because people should be cautious that there may be risks of using KD by healthy users and overuse should be avoided (Schugar & Crawford, 2012). In the last decade, several studies investigated effects of KD on naive animals, however, findings are quite contradictory. For example, Zhao, Stafstrom, Fu, Hu, and Holmes (2004) reported KD have detrimental effects on spatial learning memory and brain growth, while the other study claimed its beneficial effects in novel object recognition test (Brownlow, Jung, Moore, Bechmann, & Jankord, 2017). Many studies even failed to find its influence on cognitive function (Thio et al., 2010; Fukushima et al., 2015). Electrophysiologically, several studies demonstrated impaired long‐term potentiation (LTP) by KD in freely behaving rats (Koranda, Ruskin, Masino, & Blaise, 2011; Blaise, Ruskin, Koranda, & Masino, 2015), despite of that the other find negative results (Thio et al., 2010). In addition, KD is shown to increase sociability in young male rats (Kasprowska‐Liskiewicz et al., 2017). However, it exhibited minimal effect in other studies (Ruskin et al., 2013; Verpeut, DiCicco‐Bloom, & Bello, 2016; Castro, Baronio, Perry, Riesgo, & Gottfried, 2017). The reasons for these inconsistencies are not very clear, possibly due to variation in animal husbandry and/or experimental designs. Moreover, most of earlier studies employed short‐term feeding strategy, ranging from 2 weeks to 2 months, to examine the effect of KD. It is not known whether long‐term feeding of KD would show more profound effect on physiological behaviors.

In this study, we conducted a series of behavioral tests and electrophysiological recordings on male adult naive mice fed with KD for 3 months to investigate the effect of KD on physiological behaviors and synaptic functions.

## 2 MATERIALS AND METHODS

### 2.1 Animals

Adult male C57BL/6J mice at age of 2–3 months were purchased from Guangdong Medical Laboratory Animal Center. Animals were group‐housed and allowed to acclimate to the facility for 1 week prior to experiments. Throughout experimental procedure, mice were single‐housed in a room with ad libitum access to food and water. Housing conditions were maintained at a temperature of 22 ± 1°C, at >30% humidity and a standard 12 hr light/dark cycle (08:00–20:00). All experiments were performed in accordance with National Institutes of Health guide for the care and use of Laboratory animals, and approved by the Animal Ethics Committee of Guangzhou Medical University.

### 2.2 Diets and feeding

The content of customized diets (Research Diets) is as follows (per‐calorie macronutrient): control (D10070802), 10% protein, 80% carbohydrates, and 10% fat; ketogenic diet (KD, D10070801), 10% protein and 90% fat (also see Table 1). The fat sources are soybean oil and cocoa butter. Micronutrient content, fiber, and preservatives are matched according to a per‐calorie basis. Detailed description of the diet composition is shown in the Table 2. Because of stick‐like texture of control and KD, these diets were easily placed in the food well of the cage‐top wire lid. Both diets were changed weekly for all cages; Efforts were made to ensure that there was minimal to no wastage of food in the cage bottoms. Food intake and body weight were measured every other day at same time. Briefly, at ~2 p.m., food on the wire lid was measured by a weighting scale with gram as unit and precision scale of 2. Food intake per day was calculated as: (current weighting value—last weighting value)/2. The kilocalorie per gram (kcal/g) for control diet and KD are: 3.8 and 6.7, respectively (see table 1). Thus, the total caloric intake per day can be calculated as: 3.8 × food intake per day and 6.7 × food intake per day for control and KD, respectively.

**Table 1:** Percentage of three energy substances in diet

Product#	Control (D10070802)		KD (D10070801)	
	g%	kcal%	g%	kcal%
Protein	10	10	17	10
Carbohydrate	77	80	0	0
Fat	4	10	67	90
Total	—	100	—	100
kcal/g	3.8	—	6.7	—

**Table 2:** Detailed description of the diet composition

Ingredient	Control (D10070802)		KD (D10070801)	
	g%	kcal%	g%	kcal%
Casein, 80 mesh	100	400	100	400
l‐Cystine	1.5	6	1.5	6
Com starch	371	1,484	0	0
Maltodestrin 10	35	140	0	0
Sucrose	406	1624	0	0
Cellulose, BW200	50	0	50	0
Soybean oil	25	225	25	225
Cocoa butter	20	180	381	3,429
Mineral mix, S10026	10	0	10	0
Dicalcium phosphate	13	0	13	0
Calcium carbonate	5.5	0	5.5	0
Potassium citrate, 1 H_2_O	16.5	0	16.5	0
Vitamin mix,V10001C, 10 × Vits	1	0	1	0
Choline bitartrate	2	0	2	0
FD&C yellow dye #5	0.025	0	0.025	0
FD&C red dye #40	0	0	0.025	0
FD&C blue dye #1	0.025	0	0	0
Total	1,056.6	4,059	605.55	4,060

### 2.3 Blood ketones

Blood ketone levels were measured using the blood glucose and ketone monitoring system (FreeStyle Optium Neo, Abbott) according to the manufacturer's instructions. The blood was collected between 2 and 3 p.m. on the measuring day. Briefly, after sterilized with 70% ethanol, the tail tips of mice under test were cut by a clean scissor and a drop of blood was collected. Using the test strip (Abbott), levels of β‐hydroxybutyrate were determined.

### 2.4 Behavioral analysis

All behavioral tests were conducted consecutively with an interval of 2 days, to minimize the interference of each test on the others. All tests were performed between 1  and 5 p.m. on the test day. Mice were handled by test performers for 3 days before behavioral tests. On the test day, mice were acclimated to the testing room for 1 hr before testing. Locomotor activity was measured as described previously (Sun et al., 2016). Briefly, mice were placed in a chamber (40 × 40 × 20 cm) and movement was monitored for 30 min using an overhead camera and tracking software (SMART 3.0, Panlab). The center 20 × 20 cm region was artificially defined as the center region. Number of entries and duration spent in the center region were recorded.

Elevated plus maze (EPM) test was performed under illumination of 200 Lux, starting from 1 p.m. on the test day. The EPM, consisting of two opposing wall‐closed arms and two open arms, each at 5 × 30 cm, was placed ~50 cm above the floor. Mice were gently placed in the central area with its nose facing one of the closed arms. Their movement was recorded for 5 min using an overhead camera and tracking software (SMART 3.0, Panlab). The time that mice spent in the open arms, closed arm, center area, and the number of entries were quantified autonomously. The percentage (%) of time that mice spent in a given area was defined as time spent in the given area (sec)/the total time (300 s) × 100.

During Y‐maze test, mice were placed at the center of a Y‐shaped maze with three arms (35 cm) at an angle of 120°, and allowed to move freely through the maze for 8 min. The total number and series of arm entries were recorded. Nonoverlapping entrance sequences (e.g., ABC, BCA) were defined as spontaneous alternations. The percentage of spontaneous alternation was defined as the ratio of total number of spontaneous alternations to (total arm entries‐2)  × 100.

Morris water maze test was performed as described previously (Sun et al., 2016; Ou et al., 2018). Briefly, a day before training, mice were placed in a pool (120 cm in diameter) and scored for ability to find the visible platform (10 cm in diameter) within 60 s. Mice that failed to locate and climb onto the platform twice were excluded from further test. The platform was then moved to a new location and submerged 1 cm beneath surface of white water. Mice were trained for 5 days with 4 trials (60 s in duration) per day to climb on the hidden platform. Seven positions were used to ensure that visual spatial memory was used by mice to find the hidden platform. On the 6th day, the platform was removed and mice were placed into the pool at a new start position and scored for time spent in platform area (N30: 30 cm as diameter) and number of platform crossings within 60 s.

A clear glass cylinder at a height of 40 cm and diameter of 12 cm was used for the forced swimming test (FST). During the test, the cylinder was filled with water (22 ± 1°C) to 23 cm. Mice were gently put into the cylinder recorded for 6 min. The duration of their immobility was quantified autonomously by software (SMART 3.0, Panlab).

For tail suspension test, a mouse was hanged by wrapping the tail with adhesive tape onto a hook, which connects to the ceiling of the rectangular compartment (70 cm in height, 30 cm in width, 30 cm in depth), and video‐recorded for 6 min. The duration of immobility was quantified autonomously by software (SMART 3.0, Panlab).

The social interaction test was performed as described previously with minor modification (Mei et al., 2016; Wang et al., 2018). Briefly, a rectangular apparatus consisted of two chambers (20 × 25 × 25 cm) with a neutral middle zone (12 × 25 × 25 cm) that allowed for unbiased entry into either chamber. During phase I test, mice were put into the middle zone and habituated for 5 min. An unfamiliar mouse (Stranger 1, S1) was then introduced into a wire cage in left chamber and an empty wire cage put on the right chamber. The test mouse was allowed to freely explore all three chambers for 5 min. The animal remains in the chamber for an extra 5 min to better acquire cues from S1 mouse. During phase II test, a novel stranger mouse (Stranger 2, S2) was put into the previously empty wire cage and the test animal was allowed to explore for another 5 min. Time spent in each chamber was calculated by SMART 3.0 software (Panlab).

For the rotarod test, mice were placed on the rotarod, facing away from the direction of rotation. The rotarod was set with a start speed of 4 rpm, accelerating gradually to 40 rpm in 5 min. Mice were trained twice to adapt to the rod. Briefly, mice were repeatedly placed on the rod if they fall down, until the 5‐min session ends. After rest of 1.5 hr, mice were placed on the rod again, rotating with a speed of 4 rpm to 40 rpm to record the latency when mice fall and speed at which mice fall. Tests were repeated three times on the same day, with interval time at ~1.5 hr. Mean speed at fall was defined as (4 + *S*)/2, where *S* is the speed at which mice fall.

To measure the seizure susceptibility, mice were injected with PTZ at dose of 50 mg/kg (i.p.), as previously described (Sun et al., 2016). Right after PTZ injection, mice were put back into their home cages and the time was recorded. The latency till the onset of generalized convulsive seizures (GS) was recorded. Behavioral seizures were scored based on the criteria by Racine (Racine, 1972): stage 0, no seizure; stage 1, head nodding; stage 2, sporadic full‐body shaking, spasms; stage 3, chronic full‐body spasms; stage 4, jumping, shrieking, falling over; and stage 5, violent convulsions, falling over, death. Stages 4 and 5 were considered as GS. If GS was not observed in 20 min, 20 min were scored.

### 2.5 Electrophysiological recording

Hippocampal slices were prepared as described previously (Lu et al., 2014). Briefly, mice were anesthetized with isoflurane and brains were extracted and chilled in ice‐cold modified artificial cerebrospinal fluid (ACSF) which contains (in mM): 220 Sucrose, 2 KCl, 10 MgSO_4_, 0.2 CaCl_2_, 1.3 NaH_2_PO_4_, 26 NaHCO_3_, and 10 glucose. Coronal hippocampal slices (300 µm) were cut in ice‐cold modified ACSF with a VT‐1000S vibratome (Leica, Germany) and transferred to an incubation chamber containing regular ACSF (in mM) (126 NaCl, 3 KCl, 1 MgSO_4_, 2 CaCl_2_, 1.25 NaH_2_PO_4_, 26 NaHCO_3_, and 10 glucose) at 32°C for 30 min and at room temperature (25 ± 1°C) for additional 1 hr before recording. All solutions were saturated with 95% O_2_/5% CO_2_ (vol/vol).

Slices were placed in the recording chamber, which was superfused (2 ml/min) continuously with ACSF. Slices were visualized with infrared optics using an upright microscope equipped with an infrared‐sensitive CCD camera (DAGE‐MTI, IR‐1000E). The pipettes were pulled by a micropipette puller (P‐97, Sutter instrument) with a resistance of 3–5 MΩ. Recordings were made with a MultiClamp 700B amplifier and 1,440A digitizer (Molecular Device), data were filtered at 1 kHz, and sampled at 10 kHz.

The Schaffer Collaterals (SC)‐CA1 pathway was stimulated with a concentric bipolar electrode (FHC), and field excitatory postsynaptic potentials (fEPSPs) were recorded in current‐clamp with ACSF‐filled glass pipettes (1–5 MΩ). Monophasic 100‐µs pulses of constant currents with intensity was used as stimuli and adjusted to produce ~30% of maximal amplitudes, at a frequency of 0.033 Hz. The strength of synaptic transmission was determined by measuring the initial (10%–60% rising phase) slope of fEPSPs. Long‐term potentiation was induced by two trains of 100 pulses in 1 sec, at an interval of 20 s. The level of LTP was determined at an average of 50–60 min after tetanus stimulation.

### 2.6 Statistical analysis

Statistical analysis was done by the GraphPad Prism version 5.0 (GraphPad Software). Complete results of the statistical analysis, including degrees of freedom and exact *p‐*values are presented in results section and figure legends. Sample size choice was based on previous studies (Lu et al., 2014; Sun et al., 2016), not predetermined by a statistical method. No randomization method was used. The normality of data was tested by Shapiro–Wilk normality test. No specific statistical corrections were used. A two‐way ANOVA was used in analysis of input‐output relationship and social interaction results. A repeated two‐way ANOVA was used in body weight and calorie measurement, water maze test. The least significant difference (LSD) was used for *post hoc* comparisons. A Student's *t* test was used to compare data from two groups. All tests were two‐sided. Data are represented as mean ± SEM. Value of *p* < 0.05 was considered to be statistically significant.

## 3 RESULT

### 3.1 Increased blood ketone level and decreased body weight by ketogenic diet

**Figure 1:**
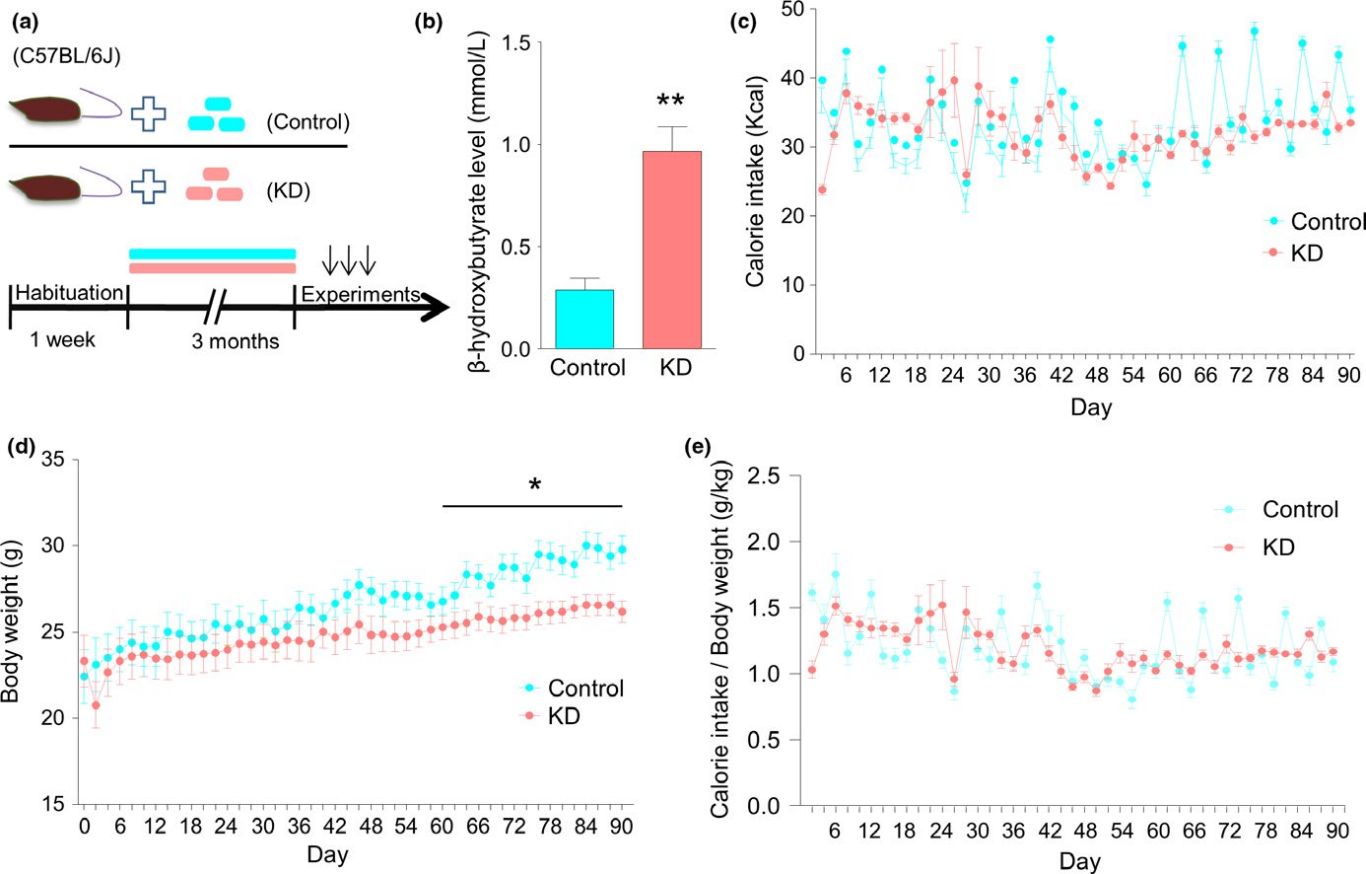
Ketogenic diet (KD) increases blood ketone level and decreases body weight. (a) Diagram of experimental design. After 1 week of habituation, mice were divided into two groups, fed with control and KD, respectively, for 3 months. Subsequent experiments were then performed. (b) Increased circulating levels of β‐hydroxybutyrate after 3 months of KD treatment. *n* = 9 mice per group. Student's *t* test, **p < 0.01. (c) Comparable caloric intake between the control and KD groups. Caloric intake was measured by weighting food every other day. *n* = 9 mice per group. Repeated two‐way ANOVA, *F*(1,704) = 3.744, *p* = 0.0709. (d) Decreased weight gain in KD‐fed mice. Body weight was measured every other day. *n* = 9 mice per group. Repeated two‐way ANOVA, for the whole period, *F*(1,720) = 2.115, *p* = 0.1652; For the last month, *F*(1,240) = 7.44, *p = 0.0149. n.s. denotes not significant, “*” denotes significant. (e) Similar caloric intake per kg of body weight between the two groups. *n* = 9 mice per group. Repeated two‐way ANOVA, *F*(1,704) = 0.0306, *p* = 0.8633

C57BL/6J adult male mice (2–3 months) were divided into two groups, and fed with control diet or KD (Figure 1a). After 3 months, the concentration of β‐hydroxybutyrate, one major form of ketone, was observed dramatically increased in the KD group, and compared with controls (Figure 1b, Student's *t* test, *t*(16) = 5.066, **p = 0.0001), indicating that KD successfully increased ketone production, as expected. During the feeding period, caloric intake and body weight were measured every other day. As shown in Figure 1c, the level of caloric intake was comparable between the control and KD groups throughout the feeding period (Repeated two‐way ANOVA, *F*(1,704) = 3.744, *p* = 0.0709). Body weight in both the groups was increased with time. Although the weight gain was not different between the two groups during the whole period, it was much slower in the KD group compared with control during the last month (Figure 1d, repeated two‐way ANOVA, for the whole period *F*(1,720) = 2.115, *p* = 0.1652; for the last month, *F*(1,240) = 7.44, *p = 0.0149.). In addition, we analyzed the daily caloric intake per kg of body weight. Still, there was no difference between the two groups (Figure 1e, repeated two‐way ANOVA, *F*(1,704) = 0.0306, *p* = 0.8633).

### 3.2 KD increases seizure threshold without alterations of locomotor activity and anxiety level

**Figure 2:**
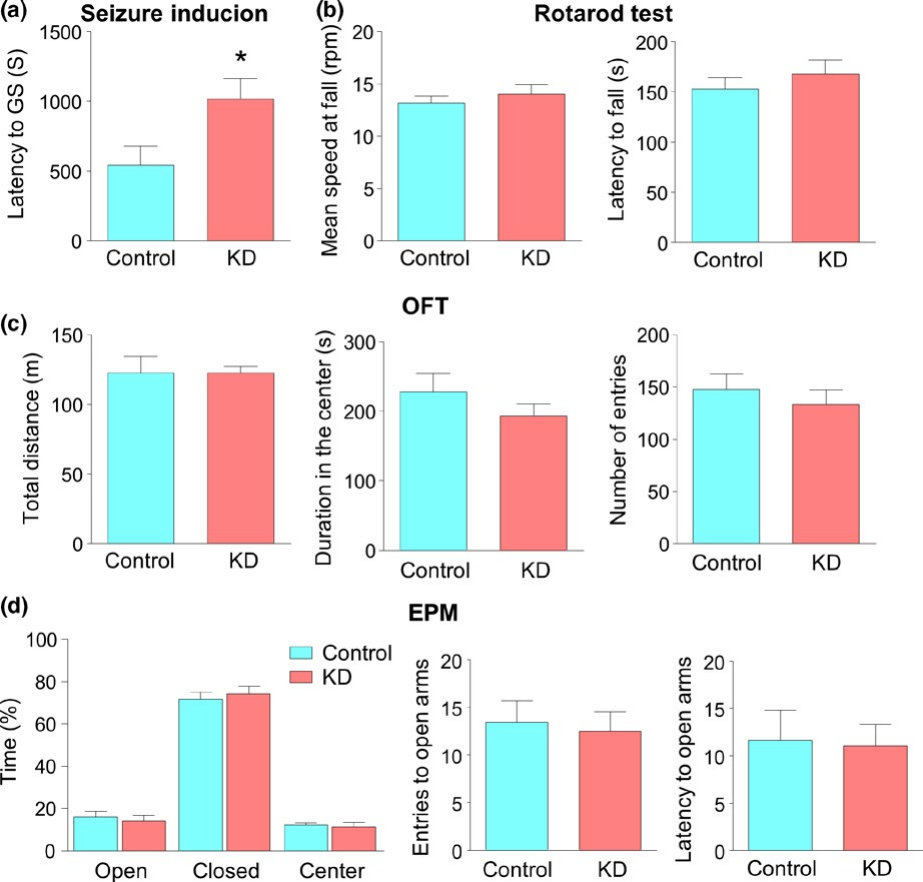
Ketogenic diet (KD) increases seizure threshold without alteration of locomotor activity and anxiety level. (a) Increased latency of mice fed with KD to generalized convulsive seizure in response to PTZ. *n* = 6 mice for the control group, *n* = 5 mice for the KD group. Student's *t* test, *t*(9) = 2.381, *p = 0.0412. “*” denotes significant. (b) Similar mean speed while mice fall in the rotarod test (left) and latency to fall in the rotarod test (right). Mean speed at fall was defined as (4 + *S*)/2, where *S* is the speed at which mice fall. *n* = 9 mice for each group. Student's *t* test, for left, *t*(16) = 0.797, *p* = 0.4371; for right, *t*(16) = 0.8212, *p* = 0.4236. (c) Similar total distance traveled (left), comparable duration that mice spent in the center (middle) and similar number of entries to the center in the open field test (OFT) (right). *n* = 9 mice per group. Student's *t* test, for left, *t*(16) = 0.0159, *p* = 0.9875; for middle, *t*(16) = 1.088, *p* = 0.2926; for right, *t*(16) = 0.6866, *p* = 0.5021. (d) No difference in duration in the open arms (left), number of entries to open arms (middle) and latency of the first entry to open arm in elevated plus maze test (EPM) (right). *n* = 9 mice per group. Student's *t* test, for left, *t*(16) = 0.4532, *p* = 0.6565; for middle, *t*(16) = 0.3243, *p* = 0.7499; for right, *t*(16) = 0.1556, *p* = 0.8783

It is well known that KD is an effective treatment for refractory epilepsy, as demonstrated by numerous studies from clinical and animal experiments (van der Louw et al., 2016; Youngson et al., 2017). To examine whether long‐term feeding of KD affects seizure susceptibility, Mice were intraperitoneally injected with pentylenetetrazol (PTZ), a GABAa receptor antagonist, which was frequently used to induce seizure (Suzuki et al., 2009; Dhir, 2012). The latency to the onset of generalized convulsive seizures (GS) was much higher in mice of the KD group than those in the control group (Figure 2a, Student's *t* test, *t*(9) = 2.381, *p = 0.0412). This result suggests that KD decreased seizure susceptibility, which is in agreement with previous reports (Todorova, Tandon, Madore, Stafstrom, & Seyfried, 2000; Mantis, Centeno, Todorova, McGowan, & Seyfried, 2004; Maalouf, Rho, & Mattson, 2009).

Because of slower weight gain in KD‐fed mice, we suspected that KD may affect their motor capacity. In fact, it was reported that KD is able to improve the motor coordination and cognition recovery in young rats suffering from traumatic brain injury (Appelberg, Hovda, & Prins, 2009). To verify this hypothesis, we first examined the motor coordination by the rotarod test, a performance test based on a rotating rod with forced motor activity being applied. The length of time that a given animal stays on this rotating rod at accelerating speed reflects its capacity to balance and coordinate (Deacon, 2013). As shown in Figure 2b, the mean speed at mice falling and the latency to fall were similar between the two groups, suggesting that KD does not have effect on motor coordination (Student's *t* test, for mean speed at fall, *t*(16) = 0.797, *p* = 0.4371; for latency to fall, *t*(16) = 0.8212, *p* = 0.4236). We further tested the locomotor activity in the open field test (OFT), which is usually used in studies of the neurobiological basis of locomotor activity and anxiety (Kraeuter, Guest, & Sarnyai, 2019). The total distance of mice in the KD group that travelled in 30 min was not different from that in the control group (Figure 2c, Student's *t* test, *t*(16) = 0.0159, *p* = 0.9875). Taken together, these observations suggest that motor function is not altered by KD. We also measured the duration in the center and the number that mice enter the center in OFT. Both were comparable between the two groups (Figure 2c, Student's *t* test, for duration in the center, *t*(16) = 1.088, *p* = 0.2926; for number of entry to the center, *t*(16) = 0.6866, *p* = 0.5021), indicating the similar anxiety level in both the groups. To further test this notion, we performed elevated plus maze (EPM), a classical method to evaluate the anxiety level (Belzung & Griebel, 2001). As shown in the Figure 2d, the percentage of duration that mice with control diet spent in open arms was not different from that of mice with KD (Student's *t* test, *t*(16) = 0.4532, *p* = 0.6565). In addition, the number of entries to open arms and the latency to open arm were not changed either (Figure 2d, Student's *t* test, for number of entries to open arms, *t*(16) = 0.3243, *p* = 0.7499; for latency to open arm, *t*(16) = 0.1556, *p* = 0.8783), in agreement with results from OFT. In short, anxiety level in adult naive mice is not affected by consumption of KD.

### 3.3 No effect of KD on spatial memory

**Figure 3:**
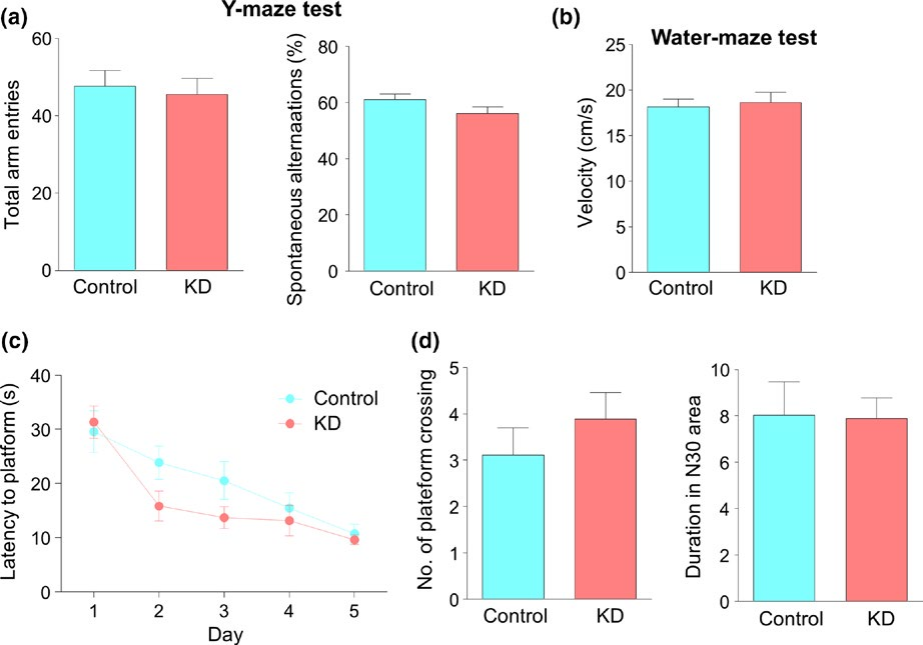
No effect of ketogenic diet (KD) on spatial memory. (a) Similar total arm entries (left) and levels of spontaneous alternations (right) between the two groups in Y‐maze test. *n* = 9 mice per group. Student's *t* test, for left, *t*(16) = 0.3593, *p* = 0.7241; for right, *t*(16) = 1.642, *p* = 0.1201. (b) Comparable velocity in Morris water maze of the two groups. *n* = 9 mice per group. Student's *t* test, *t*(16) = 0.3389, *p* = 0.7391. (c) Comparable latency to the platform between the two groups during the hidden platform task in the water maze test. *n* = 9 mice per group. Repeated two‐way ANOVA, *F*(1,80) = 3.547, *p* = 0.0633. (d) No difference in the number of platform crossings (left) and the time in the N30 area during test. N30 area was defined as a circle with 30 cm in diameter and shares the same center with the platform. *n* = 9 mice per group. Student's *t* test, for left, *t*(16) = 0.9548, *p* = 0.3539; for right, *t*(16) = 0.0846, *p* = 0.9336

To examine whether KD has an influence on cognitive function, we first measured their spatial recognition memory, a kind of short‐term memory, by the Y‐maze test which is based on the innate tendency of mice to explore novel environments (Dudchenko, 2004; Jiao et al., 2017). Mice in the KD group exhibited comparable number of arm entries to those in the control group (Figure 3a, Student's *t* test, *t*(16) = 0.3593, *p* = 0.7241), in agreement with the result of no change in locomotor activity. The rate of spontaneous alterations was also similar between the two groups (Figure 3a, Student's *t* test, *t*(16) = 1.642, *p* = 0.1201). These results suggest that KD has no effect on spatial recognition memory. To further characterize the effects of KD on long‐term memory, mice were subjected to Morris water maze test, a classical behavioral paradigm to examine spatial learning and memory consolidation (Morris, Garrud, Rawlins, & O'Keefe, 1982). Mice in the KD group displayed similar swimming velocity to those in the control group (Figure 3b, Student's *t* test, *t*(16) = 0.3389, *p* = 0.7391). During the training phase, the escape latency for the KD group to locate the hidden platform was similar to that for the control group on the first day. Interestingly, on the second and third days, mice in the KD group spent less time to locate the platform, suggesting a faster learning. However, during the last two days, the two groups eventually showed similar latency to find the platform (Figure 3c, repeated two‐way ANOVA, *F*(1,80) = 3.547, *p* = 0.0633). In the probe test when the platform had been removed, mice in the two groups exhibited similar crosses over the absent platform and spent comparable time in the 30‐cm area (N30) surrounding the absent platform (Figure 3d, Student's *t* test, for number of platform crossing, *t*(16) = 0.9548, *p* = 0.3539; for duration in N30 area, *t*(16) = 0.0846, *p* = 0.9336). These results suggest that although KD has a minor positive effect on learning ability, it shows no influence on spatial memory consolidation.

### 3.4 Unaltered social interaction and depressive‐like behaviors by KD

**Figure 4:**
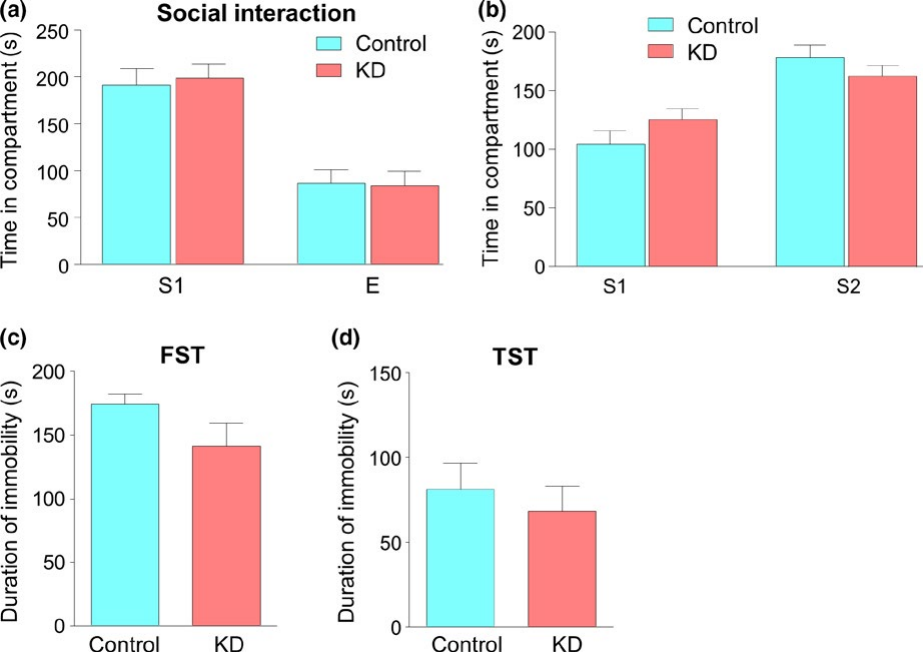
Unaltered social interaction and depressive‐like behaviors by ketogenic diet (KD). (a) KD‐fed mice showed similar level of preference to control for social interaction with a stranger (S1) mouse rather than an empty cage (E). *n* = 8 mice per group. Two‐way ANOVA, *F*(1,28) = 0.0106, *p* = 0.9186. (b) Similar level of preference for social interaction with a stranger (S2) mouse rather than the previous S1 mouse. *n* = 8 mice per group. Two‐way ANOVA, *F*(1,28) = 0.1307, *p* = 0.7204. (c) Comparable immobility time in the forced swimming test (FST) between the two groups. *n* = 9 mice per group. Student's *t* test, *t*(16) = 1.645, *p* = 0.1195. (d) No difference in the immobility time in the tail suspension test (TST) between the two groups. *n* = 9 mice per group. Student's *t* test, *t*(16) = 0.605, *p* = 0.5537

It has been reported that KD has a beneficial effect on sociability of patients and animal models of autism spectrum disorder, as well as naive young rats (Ahn, Narous, Tobias, Rho, & Mychasiuk, 2014; Kasprowska‐Liskiewicz et al., 2017). However, whether KD, especially in the case of long‐term intervention, exhibits a similar effect on adult male mice is not clear. We utilized a modified three‐chamber assay to test voluntary social interaction (Mei et al., 2016). After habituation to the three‐chamber box, mice were given a choice of either interacting with an empty wire cage or a stranger mouse. Time that mice spent in either compartment was recorded. As shown in Figure 4a, mice in the KD group exhibited similar preference for the stranger mouse (S1) over the empty wire cage to that in the control group (Two‐way ANOVA, *F*(1,28) = 0.0106, *p* = 0.9186). Furthermore, while a second stranger mouse (S2) was placed into the previously empty wire cage, mice in the two groups still showed comparable preference for S2 over S1 (Figure 4b, two‐way ANOVA, *F*(1,28) = 0.1307, *p* = 0.7204). These results suggest that long‐term feeding of KD displayed no effect on social behaviors of adult naive mice.

We further investigated the effect of KD on depression‐like behaviors by using forced swimming test (FST) and tail suspension test (TST), two classical methods to detect acute behavioral despair, which is a major symptom of depression (Arauchi & Hashioka, 2018). As shown in Figure 4c, there was a trend that the duration of immobility for the KD group was a little shorter than controls, although it was not statistically significant (Student's *t* test, *t*(16) = 1.645, *p* = 0.1195). Similar result was also found in TST (Figure 4d, Student's *t* test, *t*(16) = 0.605, *p* = 0.5537). Together with the results of no change in anxiety level, these observations suggest that long‐term intervention of KD may have minimal effect on emotion state of adult naive mice.

### 3.5 Normal basal synaptic transmission and synaptic plasticity by KD

**Figure 5:**
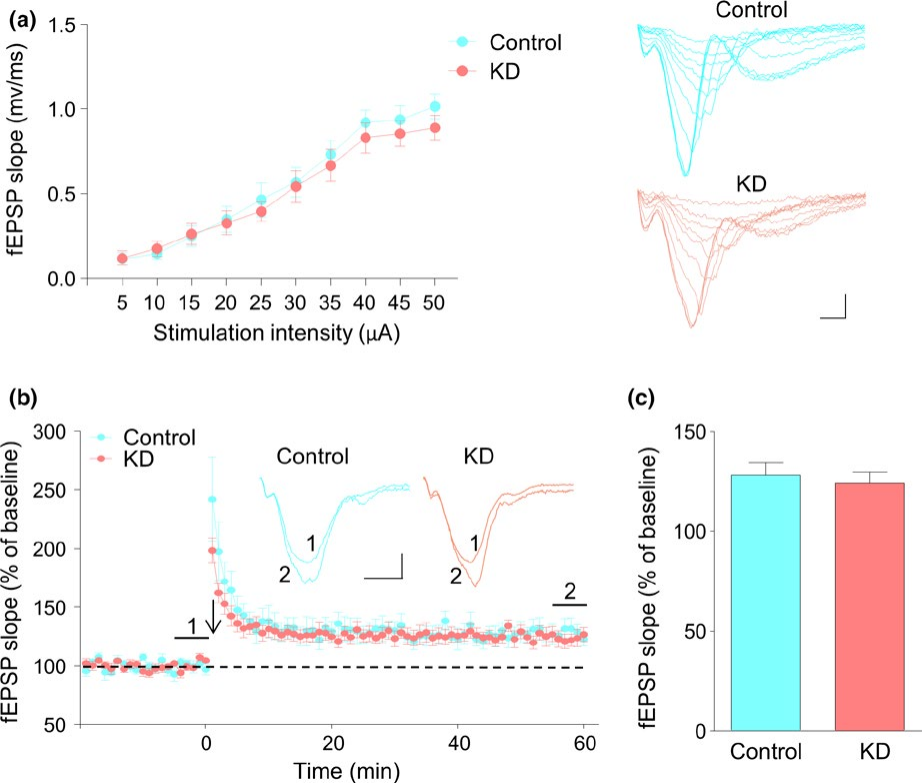
No effect of ketogenic diet (KD) on basal synaptic transmission and synaptic plasticity. (a) Similar amplitudes of fEPSP slopes. fEPSPs were recorded in the hippocampus with stimulation of the SC‐CA1 pathway at gradual increasing intensities. *n* = 9 slices from 3 mice for each group. Two‐way ANOVA, *F*(1,160) = 1.75, *p* = 0.1877. Shown on the right are representative fEPSP traces. Scale bars: 20 ms, 0.5 mV. (b) Normal LTP at SC‐CA1 synapses in the hippocampus of mice fed with KD. Normalized fEPSP slopes were plotted every 1 min. Arrow denotes LTP induction. Shown on the right are representative traces taken before (1) and 50 min after tetanic stimulation (2). Scale bars: 5 ms, 0.2 mV. (c) Quantitative analysis of LTP level in (b). *n* = 9 slices from 3 mice for each group. Student's *t* test, *t*(16) = 0.7804, *p* = 0.4466

Increased threshold to seizure by KD may indicate altered synaptic transmission efficiency. To demonstrate this notion, field excitatory postsynaptic potentials (fEPSPs) were recorded at the SC‐CA1 pathway in the hippocampus. We compared the input–output relationship and found that the fEPSP slopes of the KD group at gradually increased densities were similar to those of the control group (Figure 5a, two‐way ANOVA, *F*(1,160) = 1.75, *p* = 0.1877), These data are in agreement with a previous report (Stafstrom, Wang, & Jensen, 1999). These results suggest unaltered basal synaptic transmission. We also examined their synaptic plasticity by recording long‐term potentiation (LTP) with tetanic stimulation. As shown in Figure 5b, the induction and maintenance of LTP was not changed, with a potentiation level of 130.7% and 124.1% for the control and KD groups, respectively (Figure 5c, Student's *t* test, *t*(16) = 0.7804, *p* = 0.4466). In short, these findings suggest that KD feeding has no effect on basal synaptic transmission and plasticity in adult naive mice.

## 4 DISCUSSION

In this study, we found that, with 3‐month feeding of KD, adult naive mice displayed elevated blood ketone level and seizure threshold with slower weight gain, compared with control. However, there was no effect of KD on motor coordination and locomotor activity, anxiety level, spatial learning and memory, sociability, and depression‐like behaviors. Electrophysiological recording results indicated unaltered basal synaptic transmission and LTP in the hippocampus of KD‐fed mice.

The low‐carbohydrate and high‐fat dietary habit is attracting more and more attention, not only among researchers, but also within society, especially in the context of weight loss and sports performance (2017; Gershuni, Yan, & Medici, 2018). Thus, it is urgent to strengthen our understanding of the role of KD in disease as well as in healthy individuals. Although tremendous number of researches report the effects of KD on a variety of neurological and non‐neurological disorders, its effect on naive animals is less investigated and existing data is not conclusive. Distinct reasons may account for the discrepancy, such as strain, animal age, composition of KD, feeding strategy. Interestingly, most studies adopted short‐term feeding strategy, ranging from 2 weeks to 2 months. In addition, naive rats were frequently used to examine the effect of KD on physiological function. In contrast, very few studies used normal mice as research objects. To our knowledge, this is the first study to specially investigate the influence of long‐term feeding of KD on the young adult naive mice.

In our experiments, we successfully induced therapeutic ketosis by elevating ketone level to ~1 mM in adult naive mice with KD, which consists of 90% fat and 10% protein. Weight gain was slower in KD‐fed mice, although the caloric intake was not different from that of control mice. These results are consistent with previous reports (Thio et al., 2010; Fukushima et al., 2015; Kasprowska‐Liskiewicz et al., 2017). Slower weight gain in KD‐fed mice led us to examine the effect of KD on motor function. We found minimal change in motor coordination and locomotor activity, as evaluated in rotarod test and open field test, respectively. Interestingly, KD has been demonstrated to have beneficial effect on motor activity in aged animals (Newman et al., 2017; Roberts et al., 2017). These results suggest that the KD may function in an age‐dependent manner.

The reason for decreased weight gain under circumstance of equal caloric intake and locomotor activity is not very clear. One possible mechanism may be due to the alteration of resting energy expenditure (Gershuni et al., 2018). This could be caused by decreased insulin level and higher levels of plasma ketone bodies which induce a higher use of lipids by cells, or by changed level of hormones including glucocorticoid, which has been associated with growth suppression (Jequier, 2002; Peres, Nogueira, Paula, Costa, & Ribeiro, 2013). It should be noted that we only examined the locomotor activity of mice in the day time. It is not known whether mice fed with KD exhibit altered level of activity during the night. Further experiments are warranted in future to investigate the effect of KD on resting energy expenditure.

Results of the open field test and elevated plus maze test indicate that anxiety is not altered by KD. Additionally, depression‐like behaviors, as assessed in forced swimming test and tail suspension test, were also not changed, in agreement with earlier studies (Fukushima et al., 2015; Kasprowska‐Liskiewicz et al., 2017). It is widely known that side effect of KD in clinics includes constipation, vomit, and inappetence, which frequently deteriorates one's emotion. Our observations suggest that with long‐term feeding of KD, emotion state is hardly affected in mice.

The Y‐maze and water maze tasks indicate that the learning ability, short‐ and long‐term memory are not impaired by KD. In contrast, it seems that KD‐fed mice actually learn faster than controls, although it shows only a tendency without statistical significance. However, this is not consistent with a previous study showing that the KD impaired performance on the Morris water maze test (Zhao et al., 2004). One possible reason is difference in feeding period. In their study, KD was fed on immature rats starting from P21 to P60, a developmental stage when neuronal circuit is still in the process of modification and maturation. However, young adult mice, in which the brains were considerably developed, were utilized in our study to dissect the effect of KD. Consistent with results from Y‐maze and water maze tasks, synaptic plasticity in the hippocampus was not altered either.

We also tested the social behaviors of KD‐fed mice with three‐chamber apparatus and found no change in the social exploration preference, suggesting the social behavior is little affected by long‐term feeding of KD. These results are interesting in light that numerous studies have reported beneficial effect of KD in social behaviors in autism patients, animal models of autism and wildtype young adult rats (Ruskin et al., 2013; Ahn et al., 2014; Verpeut et al., 2016; Castro et al., 2017; Kasprowska‐Liskiewicz et al., 2017). The real reason for the discrepancy is not clear. A proposed mechanism is that the beneficial effect of KD is age‐dependent. In the literature, animals at age of 3 weeks or 1 month were fed with KD, feeding process lasted 10 days or 1 month. However, in our study, mice for experiments were considered adult, a developed stage when neuronal plasticity is much different from that of young mice. Another reason may be due to the context of social test. It has been reported that social interaction test carried out in the home cage of the tested animal is more sensitive than three‐chamber test (Kasprowska‐Liskiewicz et al., 2017). It will be interesting to verify whether the effect of KD on sociability is age‐ and context‐dependent in future studies.

Interestingly, mice with KD exhibited dramatically elevated seizure induction threshold, in accordance with numerous studies showing that application of the KD to multiple animal epilepsy models has demonstrated therapeutic effects, including increased induced‐seizure threshold, delayed seizure development, attenuated seizure risk and decreased the seizure severity (Todorova et al., 2000; Mantis et al., 2004; Maalouf et al., 2009; Kawamura, Ruskin, & Masino, 2016). A number of mechanical studies suggest potential therapeutic mechanisms of KD, including metabolic effects of ketonemia, decreased blood glucose and insulin levels; neuronal effects involving ATP‐sensitive potassium (KATP) channel modulation, enhanced purinergic signaling, glutamatergic and/or GABAergic neurotransmission, increased brain‐derived neurotrophic factor (BDNF) expression, attenuation of neuroinflammation, as well as improved mitochondrial function, indirect effect through gut microbiota (Boison, 2017; Puchalska & Crawford, 2017; Youngson et al., 2017; Augustin et al., 2018). Further investigations of the mechanisms underlying the specific effect of KD on epilepsy susceptibility rather than other behaviors in naive mice are warranted.

In summary, we examined the effect of long‐term feeding of KD on adult naive mice with a bunch of behavioral tests and electrophysiological recordings. Results indicate that KD exhibits little effect in naïve mice, except alteration of weight gain and seizure threshold. These observations suggest that KD may function most probably in the condition of behavioral impairments occurring in diseases. Our study contributes to the better understanding of effects of the KD on physiological behaviors and synaptic functions.

## CONFLICT OF INTEREST

The authors declare no conflict of interest.

## References

[brb31246-bib-0001] Ahn, Y. , Narous, M. , Tobias, R. , Rho, J. M. , & Mychasiuk, R. (2014). The ketogenic diet modifies social and metabolic alterations identified in the prenatal valproic acid model of autism spectrum disorder. Developmental Neuroscience, 36, 371–380. 10.1159/000362645 25011527

[brb31246-bib-0002] Appelberg, K. S. , Hovda, D. A. , & Prins, M. L. (2009). The effects of a ketogenic diet on behavioral outcome after controlled cortical impact injury in the juvenile and adult rat. Journal of Neurotrauma, 26, 497–506. 10.1089/neu.2008.0664 19231995 PMC2843134

[brb31246-bib-0003] Arauchi, R. , & Hashioka, S. (2018). Gunn rats with glial activation in the hippocampus show prolonged immobility time in the forced swimming test and tail suspension test. Brain and Behavior, 8, e01028 10.1002/brb3.1028 29953737 PMC6085916

[brb31246-bib-0004] Augustin, K. , Khabbush, A. , Williams, S. , Eaton, S. , Orford, M. , Cross, J. H. , … Williams, R. S. B. (2018). Mechanisms of action for the medium‐chain triglyceride ketogenic diet in neurological and metabolic disorders. The Lancet Neurology, 17, 84–93. 10.1016/s1474-4422(17)30408-8 29263011

[brb31246-bib-0005] Belzung, C. , & Griebel, G. (2001). Measuring normal and pathological anxiety‐like behaviour in mice: A review. Behavioural Brain Research, 125, 141–149. 10.1016/s0166-4328(01)00291-1 11682105

[brb31246-bib-0006] Benjamin, J. S. , Pilarowski, G. O. , Carosso, G. A. , Zhang, L. , Huso, D. L. , Goff, L. A. , … Bjornsson, H. T. (2017). A ketogenic diet rescues hippocampal memory defects in a mouse model of Kabuki syndrome. Proceedings of the National Academy of Sciences of the United States of America, 114, 125–130. 10.1073/pnas.1611431114 27999180 PMC5224378

[brb31246-bib-0007] Blaise, J. H. , Ruskin, D. N. , Koranda, J. L. , & Masino, S. A. (2015). Effects of a ketogenic diet on hippocampal plasticity in freely moving juvenile rats. Physiological Reports, 3, e12411 10.14814/phy2.12411 26009636 PMC4463838

[brb31246-bib-0008] Boison, D. (2017). New insights into the mechanisms of the ketogenic diet. Current Opinion in Neurology, 30, 187–192. 10.1097/wco.0000000000000432 28141738 PMC5409832

[brb31246-bib-0009] Brownlow, M. L. , Benner, L. , D'Agostino, D. , Gordon, M. N. , & Morgan, D. (2013). Ketogenic diet improves motor performance but not cognition in two mouse models of Alzheimer's pathology. PLoS ONE, 8, e75713 10.1371/journal.pone.0075713 24069439 PMC3771931

[brb31246-bib-0010] Brownlow, M. L. , Jung, S. H. , Moore, R. J. , Bechmann, N. , & Jankord, R. (2017). Nutritional ketosis affects metabolism and behavior in Sprague‐Dawley rats in both control and chronic stress environments. Frontiers in Molecular Neuroscience, 10, 129. 28555095 10.3389/fnmol.2017.00129 PMC5430035

[brb31246-bib-0011] Castro, K. , Baronio, D. , Perry, I. S. , Riesgo, R. D. S. , & Gottfried, C. (2017). The effect of ketogenic diet in an animal model of autism induced by prenatal exposure to valproic acid. Nutritional Neuroscience, 20, 343–350. 10.1080/1028415x.2015.1133029 26856821

[brb31246-bib-0012] Deacon, R. M. (2013). Measuring motor coordination in mice. Journal of Visualized Experiments, 75, e2609 10.3791/2609 PMC3724562 23748408

[brb31246-bib-0013] Dhir, A. (2012). Pentylenetetrazol (PTZ) kindling model of epilepsy. Current Protocols in Neuroscience, 9(Unit9), 37. 23042503 10.1002/0471142301.ns0937s58

[brb31246-bib-0014] Dudchenko, P. A. (2004). An overview of the tasks used to test working memory in rodents. Neuroscience and Biobehavioral Reviews, 28, 699–709. 10.1016/j.neubiorev.2004.09.002 15555679

[brb31246-bib-0015] Fukushima, A. , Ogura, Y. , Furuta, M. , Kakehashi, C. , Funabashi, T. , & Akema, T. (2015). Ketogenic diet does not impair spatial ability controlled by the hippocampus in male rats. BrainResearch, 1622, 36–42. 10.1016/j.brainres.2015.06.016 26111645

[brb31246-bib-0016] Gershuni, V. M. , Yan, S. L. , & Medici, V. (2018). Nutritional ketosis for weight management and reversal of metabolic syndrome. Current Nutrition Reports, 7, 97–106. 10.1007/s13668-018-0235-0 30128963 PMC6472268

[brb31246-bib-0017] Hallbook, T. , Ji, S. , Maudsley, S. , & Martin, B. (2012). The effects of the ketogenic diet on behavior and cognition. Epilepsy Research, 100, 304–309. 10.1016/j.eplepsyres.2011.04.017 21872440 PMC4112040

[brb31246-bib-0018] Jequier, E. (2002). Leptin signaling, adiposity, and energy balance. Annals of the New York Academy of Sciences, 967, 379–388. 10.1111/j.1749-6632.2002.tb04293.x 12079865

[brb31246-bib-0019] Jiao, H. F. , Sun, X. D. , Bates, R. , Xiong, L. , Zhang, L. , Liu, F. , … Mei, L. (2017). Transmembrane protein 108 is required for glutamatergic transmission in dentate gyrus. Proceedings of the National Academy of Sciences of the United States of America, 114, 1177–1182. 10.1073/pnas.1618213114 28096412 PMC5293068

[brb31246-bib-0020] Kashiwaya, Y. , Bergman, C. , Lee, J. H. , Wan, R. , King, M. T. , Mughal, M. R. , … Veech, R. L. (2013). A ketone ester diet exhibits anxiolytic and cognition‐sparing properties, and lessens amyloid and tau pathologies in a mouse model of Alzheimer's disease. Neurobiology of Aging, 34, 1530–1539. 10.1016/j.neurobiolaging.2012.11.023 23276384 PMC3619192

[brb31246-bib-0021] Kasprowska‐Liskiewicz, D. , Liskiewicz, A. D. , Nowacka‐Chmielewska, M. M. , Nowicka, J. , Malecki, A. , & Barski, J. J. (2017). The ketogenic diet affects the social behavior of young male rats. Physiology & Behavior, 179, 168–177. 10.1016/j.physbeh.2017.06.007 28623167

[brb31246-bib-0022] Kawamura, M. J. , Ruskin, D. N. , & Masino, S. A. (2016). Metabolic Therapy for temporal lobe epilepsy in a dish: Investigating mechanisms of ketogenic diet using electrophysiological recordings in hippocampal slices. Frontiers in Molecular Neuroscience, 9, 112 10.3389/fnmol.2016.00112 27847463 PMC5088211

[brb31246-bib-0023] Koppel, S. J. , & Swerdlow, R. H. (2018). Neuroketotherapeutics: A modern review of a century‐old therapy. Neurochemistry International, 117, 114–125. 28579059 10.1016/j.neuint.2017.05.019 PMC5711637

[brb31246-bib-0024] Koranda, J. L. , Ruskin, D. N. , Masino, S. A. , & Blaise, J. H. (2011). A ketogenic diet reduces long‐term potentiation in the dentate gyrus of freely behaving rats. Journal of Neurophysiology, 106, 662–666. 10.1152/jn.00001.2011 21613596 PMC3154820

[brb31246-bib-0025] Kraeuter, A. K. , Guest, P. C. , & Sarnyai, Z. (2019). The open field test for measuring locomotor activity and anxiety‐like behavior. Methods in Molecular Biology, 1916, 99–103. 10.1007/978-1-4939-8994-2_9 30535687

[brb31246-bib-0026] Kraeuter, A. K. , Loxton, H. , Lima, B. C. , Rudd, D. , & Sarnyai, Z. (2015). Ketogenic diet reverses behavioral abnormalities in an acute NMDA receptor hypofunction model of schizophrenia. Schizophrenia Research, 169, 491–493. 10.1016/j.schres.2015.10.041 26547882

[brb31246-bib-0027] Lu, Y. , Sun, X. D. , Hou, F. Q. , Bi, L. L. , Yin, D. M. , Liu, F. , Chen, Y. J. , Bean, J. C. , Jiao, H. F. , Liu, X. , Li, B. M. , Xiong, W. C. , Gao, T. M. , & Mei, L. (2014). Maintenance of GABAergic activity by neuregulin 1‐ErbB4 in amygdala for fear memory. Neuron, 84, 835–846. 10.1016/j.neuron.2014.09.029 25451196

[brb31246-bib-0028] Maalouf, M. , Rho, J. M. , & Mattson, M. P. (2009). The neuroprotective properties of calorie restriction, the ketogenic diet, and ketone bodies. BrainResearch Reviews, 59, 293–315. 10.1016/j.brainresrev.2008.09.002 PMC2649682 18845187

[brb31246-bib-0029] Mantis, J. G. , Centeno, N. A. , Todorova, M. T. , McGowan, R. , & Seyfried, T. N. (2004). Management of multifactorial idiopathic epilepsy in EL mice with caloric restriction and the ketogenic diet: Role of glucose and ketone bodies. Nutrition & Metabolism, 1, 11. 15507133 10.1186/1743-7075-1-11 PMC529249

[brb31246-bib-0030] Mantis, J. G. , Fritz, C. L. , Marsh, J. , Heinrichs, S. C. , & Seyfried, T. N. (2009). Improvement in motor and exploratory behavior in Rett syndrome mice with restricted ketogenic and standard diets. Epilepsy & Behavior, 15, 133–141. 19249385 10.1016/j.yebeh.2009.02.038

[brb31246-bib-0031] Mei, Y. , Monteiro, P. , Zhou, Y. , Kim, J. A. , Gao, X. , Fu, Z. , & Feng, G. (2016). Adult restoration of Shank3 expression rescues selective autistic‐like phenotypes. Nature, 530, 481–484. 10.1038/nature16971 26886798 PMC4898763

[brb31246-bib-0032] Morris, R. G. , Garrud, P. , Rawlins, J. N. , & O'Keefe, J. (1982). Place navigation impaired in rats with hippocampal lesions. Nature, 297, 681–683. 10.1038/297681a0 7088155

[brb31246-bib-0033] Newman, J. C. , Covarrubias, A. J. , Zhao, M. , Yu, X. , Gut, P. , Ng, C. P. , … Verdin, E. (2017). Ketogenic diet reduces midlife mortality and improves memory in aging mice. CellMetabolism, 26(547–557), e548. 10.1016/j.cmet.2017.08.004 PMC5605815 28877458

[brb31246-bib-0034] Ou, Z. , Kong, X. , Sun, X. , He, X. , Zhang, L. , Gong, Z. , … Xuan, A. (2018). Metformin treatment prevents amyloid plaque deposition and memory impairment in APP/PS1 mice. BrainBehavior, and Immunity, 69, 351–363. 10.1016/j.bbi.2017.12.009 29253574

[brb31246-bib-0035] Peres, R. C. , Nogueira, D. B. , de Paula, Guimaraes G. , da Costa, E. L. , & Ribeiro, D. A. (2013). Implications of ketogenic diet on weight gain, motor activity and cicatrization in Wistar rats. ToxicologyMechanisms and Methods, 23, 144–149. 10.3109/15376516.2012.735276 23038986

[brb31246-bib-0036] Poplawski, M. M. , Mastaitis, J. W. , Isoda, F. , Grosjean, F. , Zheng, F. , & Mobbs, C. V. (2011). Reversal of diabetic nephropathy by a ketogenic diet. PLoS ONE, 6, e18604 10.1371/journal.pone.0018604 21533091 PMC3080383

[brb31246-bib-0037] Puchalska, P. , & Crawford, P. A. (2017). Multi‐dimensional roles of ketone bodies in fuel metabolism, signaling, and therapeutics. CellMetabolism, 25, 262–284. 10.1016/j.cmet.2016.12.022 PMC5313038 28178565

[brb31246-bib-0038] Racine, R. J. (1972). Modification of seizure activity by electrical stimulation. II. Motor Seizure. Electroencephalography and Clinical Neurophysiology, 32, 281–294. 10.1016/j.cmet.2016.12.022 4110397

[brb31246-bib-0039] Roberts, M. N. , Wallace, M. A. , Tomilov, A. A. , Zhou, Z. , Marcotte, G. R. , Tran, D. , … Lopez‐Dominguez, J. A. (2017). A Ketogenic Diet Extends Longevity and Healthspan in Adult Mice. CellMetabolism, 26(539–546), e535. 10.1016/j.cmet.2017.08.005 PMC5609489 28877457

[brb31246-bib-0040] Ruskin, D. N. , Ross, J. L. , Kawamura, M. Jr , Ruiz, T. L. , Geiger, J. D. , & Masino, S. A. (2011). A ketogenic diet delays weight loss and does not impair working memory or motor function in the R6/2 1J mouse model of Huntington's disease. Physiology & Behavior, 103, 501–507. 10.1016/j.physbeh.2011.04.001 21501628 PMC3107892

[brb31246-bib-0041] Ruskin, D. N. , Svedova, J. , Cote, J. L. , Sandau, U. , Rho, J. M. , Kawamura, M. Jr , … Masino, S. A. (2013). Ketogenic diet improves core symptoms of autism in BTBR mice. PLoS ONE, 8, e65021 10.1371/journal.pone.0065021 23755170 PMC3673987

[brb31246-bib-0042] Schugar, R. C. , & Crawford, P. A. (2012). Low‐carbohydrate ketogenic diets, glucose homeostasis, and nonalcoholic fatty liver disease. Current Opinion in Clinical Nutrition and Metabolic Care, 15, 374–380. 10.1097/mco.0b013e3283547157 22617564 PMC3679496

[brb31246-bib-0043] Scott, J. M. , & Deuster, P. A. (2017). Ketones and Human Performance. Journal of Special Operations Medicine: A Peer Reviewed Journal for SOF Medical Professionals, 17(2), 112–116. 28599043 10.55460/PGWG-H55J

[brb31246-bib-0044] Stafstrom, C. E. , Wang, C. , & Jensen, F. E. (1999). Electrophysiological observations in hippocampal slices from rats treated with the ketogenic diet. Developmental Neuroscience, 21, 393–399. 10.1159/000017389 10575263

[brb31246-bib-0045] Sun, X.‐D. , Li, L. , Liu, F. , Huang, Z.‐H. , Bean, J. C. , Jiao, H.‐F , … Mei, L. (2016). Lrp4 in astrocytes modulates glutamatergic transmission. NatureNeuroscience, 19, 1010–1018. 10.1038/nn.4326 PMC4961622 27294513

[brb31246-bib-0046] Suzuki, T. , Miyamoto, H. , Nakahari, T. , Inoue, I. , Suemoto, T. , Jiang, B. , … Yamakawa, K. (2009). Efhc1 deficiency causes spontaneous myoclonus and increased seizure susceptibility. Human Molecular Genetics, 18, 1099–1109. 10.1093/hmg/ddp006 19147686 PMC4817086

[brb31246-bib-0047] Thio, L. L. , Rensing, N. , Maloney, S. , Wozniak, D. F. , Xiong, C. , & Yamada, K. A. (2010). A ketogenic diet does not impair rat behavior or long‐term potentiation. Epilepsia, 51, 1619–1623. 10.1111/j.1528-1167.2009.02515.x 20132289 PMC2996229

[brb31246-bib-0048] Todorova, M. T. , Tandon, P. , Madore, R. A. , Stafstrom, C. E. , & Seyfried, T. N. (2000). The ketogenic diet inhibits epileptogenesis in EL mice: A genetic model for idiopathic epilepsy. Epilepsia, 41, 933–940. 10.1111/j.1528-1157.2000.tb00275.x 10961617

[brb31246-bib-0049] van der Louw, E. , van den Hurk, D. , Neal, E. , Leiendecker, B. , Fitzsimmon, G. , Dority, L. , … Cross, J. H. (2016). Ketogenic diet guidelines for infants with refractory epilepsy. European Journal of Paediatric Neurology: Official Journal of the European Paediatric Neurology Society, 20, 798–809. 10.1016/j.ejpn.2016.07.009 27470655

[brb31246-bib-0050] Verpeut, J. L. , DiCicco‐Bloom, E. , & Bello, N. T. (2016). Ketogenic diet exposure during the juvenile period increases social behaviors and forebrain neural activation in adult Engrailed 2 null mice. Physiology & Behavior, 161, 90–98. 10.1016/j.physbeh.2016.04.001 27080080

[brb31246-bib-0051] Wang, Y. N. , Figueiredo, D. , Sun, X. D. , Dong, Z. Q. , Chen, W. B. , Cui, W. P. , … Mei, L. (2018). Controlling of glutamate release by neuregulin3 via inhibiting the assembly of the SNARE complex. Proceedings of the National Academy of Sciences of the United States of America, 115, 2508–2513. 29463705 10.1073/pnas.1716322115 PMC5877931

[brb31246-bib-0052] Youngson, N. A. , Morris, M. J. , & Ballard, J. W. O. (2017). The mechanisms mediating the antiepileptic effects of the ketogenic diet, and potential opportunities for improvement with metabolism‐altering drugs. Seizure, 52, 15–19. 10.1016/j.seizure.2017.09.005 28941398

[brb31246-bib-0053] Zhao, Q. , Stafstrom, C. E. , Fu, D. D. , Hu, Y. , & Holmes, G. L. (2004). Detrimental effects of the ketogenic diet on cognitive function in rats. Pediatric Research, 55, 498–506. 10.1203/01.pdr.0000112032.47575.d1 14711901

[brb31246-bib-0054] Zhao, Z. , Lange, D. J. , Voustianiouk, A. , MacGrogan, D. , Ho, L. , Suh, J. , … Pasinetti, G. M. (2006). A ketogenic diet as a potential novel therapeutic intervention in amyotrophic lateral sclerosis. BMC Neuroscience, 7, 29. 16584562 10.1186/1471-2202-7-29 PMC1488864

